# IRE1α inhibition by natural compound genipin on tumour associated macrophages reduces growth of hepatocellular carcinoma

**DOI:** 10.18632/oncotarget.9696

**Published:** 2016-05-30

**Authors:** Hor-Yue Tan, Ning Wang, Sai-Wah Tsao, Chi-Ming Che, Man-Fung Yuen, Yibin Feng

**Affiliations:** ^1^ School of Chinese Medicine, Li Ka Shing Faculty of Medicine, The University of Hong Kong, Hong Kong S.A.R, P.R. of China; ^2^ School of Biomedical Sciences, Li Ka Shing Faculty of Medicine, The University of Hong Kong, Hong Kong S.A.R, P.R. of China; ^3^ State Key Laboratory of Synthetic Chemistry, Chemical Biology Centre, and Department of Chemistry, The University of Hong Kong, Hong Kong S.A.R, P. R. China; ^4^ Division of Gastroenterology and Hepatology, Queen Mary Hospital and Department of Medicine, Li Ka Shing Faculty of Medicine, The University of Hong Kong, Hong Kong S.A.R, P. R. of China

**Keywords:** genipin, tumour-associated macrophage, hepatocellular carcinoma, IRE1α, NF-κB

## Abstract

Accumulating evidences postulated the influential roles of macrophages in mediating hepatocellular carcinoma (HCC) initiation and progression. In this study, we demonstrate that a small molecule, genipin reduced HCC growth through suppressing IRE1α-mediated infiltration and priming of tumour associated macrophages (TAMs). Oral administration of genipin (30mg/kg/2days) suppressed orthotopic HCC tumour growth without challenging the viability and proliferation of HCC cells. Genipin reduced infiltration of inflammatory monocytes into liver and tumour thereby suppressed TAMs presence in HCC microenvironment. Suppression of HCC growth was diminished in HCC-implanted mice with depletion of TAMs by liposome clodronate. Genipin inhibited the TAMs migration, and reduced expression of TAMs-derived inflammatory cytokines that favors HCC proliferation. This is revealed by the *in vivo* deletion of IRE1α on TAMs in genipin-treated HCC-implanted mice. Diminishing IRE1α neutralised the inhibitory effect of genipin on TAMs. Silencing the expression of IRE1α greatly reduced TAMs migration and expression of inflammatory cytokines that prime HCC proliferation. Suppression of IRE1α led to reduced XBP-1 splicing and NF-κB activation. The reduced association of IRE1α with TRAF2 and IKK complex may be responsible for the genipin-mediated inactivation of NF-κB. The findings show the important role of TAMs in inhibitory effect of genipin on HCC, and TAMs-expressing IRE1α as a promising target for disrupting the tumour environment that favor of HCC development.

## INTRODUCTION

To date, the mechanism underlying multistep hepatocellular carcinoma (HCC) progression remains ambiguous, but studies showed that inflammation may be one of the leading factors [1, 2]. Chronic inflammation re-programmes the intrahepatic environment to various tumorigenesis-promoting niches that favor cancer progenitor cell growth [3]. Recent findings proposed that the inflammatory response triggered by intestinal microbiota resulted in increased proliferation and inactivation of apoptotic signalling that promotes HCC progression [4]. Inflammation mediated HCC progression is mainly attributed by infiltration of inflammatory leukocytes into tumour microenvironment [5], and in particular, macrophages is regarded as the predominant facilitator in HCC tumour progression. Evidences showed that high population of macrophages augments HCC invasiveness, leading to poor prognosis in HCC patients [6]. The number of EGFR-expressing macrophages in HCC is correlated with HCC aggressiveness, while the high number of total macrophages is associated with low overall survival and disease free survival rate in Chinese populations [7]. These macrophages are therefore termed as tumour associated macrophages (TAMs) in some previous studies. Indeed, targeting of TAMs mediating inflammation may be potentially promising for HCC management.

Genipin is an aglycone derived from geniposide, the iridoid glycoside mainly found in the fruit of *Gardenia jasminoides* Ellis. Previous studies have postulated the potential use of genipin for treatment of osteoporosis, diabetic nephropathy, sepsis, and depression [8–11]. The anti-tumour effect of genipin is rather scanty, with a few reports on its *in vitro* cytotoxic effect in cervical and breast cancer cells [12, 13]. Our previous study has showed the inhibitory effect of genipin on intrahepatic and distant metastasis of HCC [14]. Interestingly, genipin is a well-studied anti-inflammatory metabolite [15] and many of its pharmacological activities was rooted from its anti-inflammatory action. Genipin inhibited the NLRP3 and NLRC4 inflammasome resulted in attenuation of *in vivo* peritonitis and lung inflammation in mice [16]. The suppressive effect of genipin on vascular smooth muscle cell proliferation and migration also correlated to its anti-inflammatory function [17]. But how the anti-inflammatory property of genipin contributes to its anti-tumour effect and whether TAMs is involved remains unclear.

In the present study, we investigated any inhibitory effect of genipin on orthotopic HCC implantation murine model. Simultaneously, TAMs were particularly removed with liposome clodronate injection, in order to explore the role of TAMs on tumour inhibitory effect of genipin. Bone marrow-derived macrophages were differentiated with tumour derived supernatant (TSN) to establish the *in vitro* model and the effect of genipin on viability, polarisation and priming of macrophages were studied. The involvement of IRE1α depletion in mediating inhibition of TAMs by genipin and the possible mechanisms were further elaborated. Our findings postulate the important role of inflammation in inhibitory effect of genipin on HCC, and IRE1α as a promising target for disrupting the inflammatory environment that favor of HCC development.

## RESULTS

### Genipin exhibits non-toxic suppression to the *in vivo* growth of HCC

**Figure 1:**
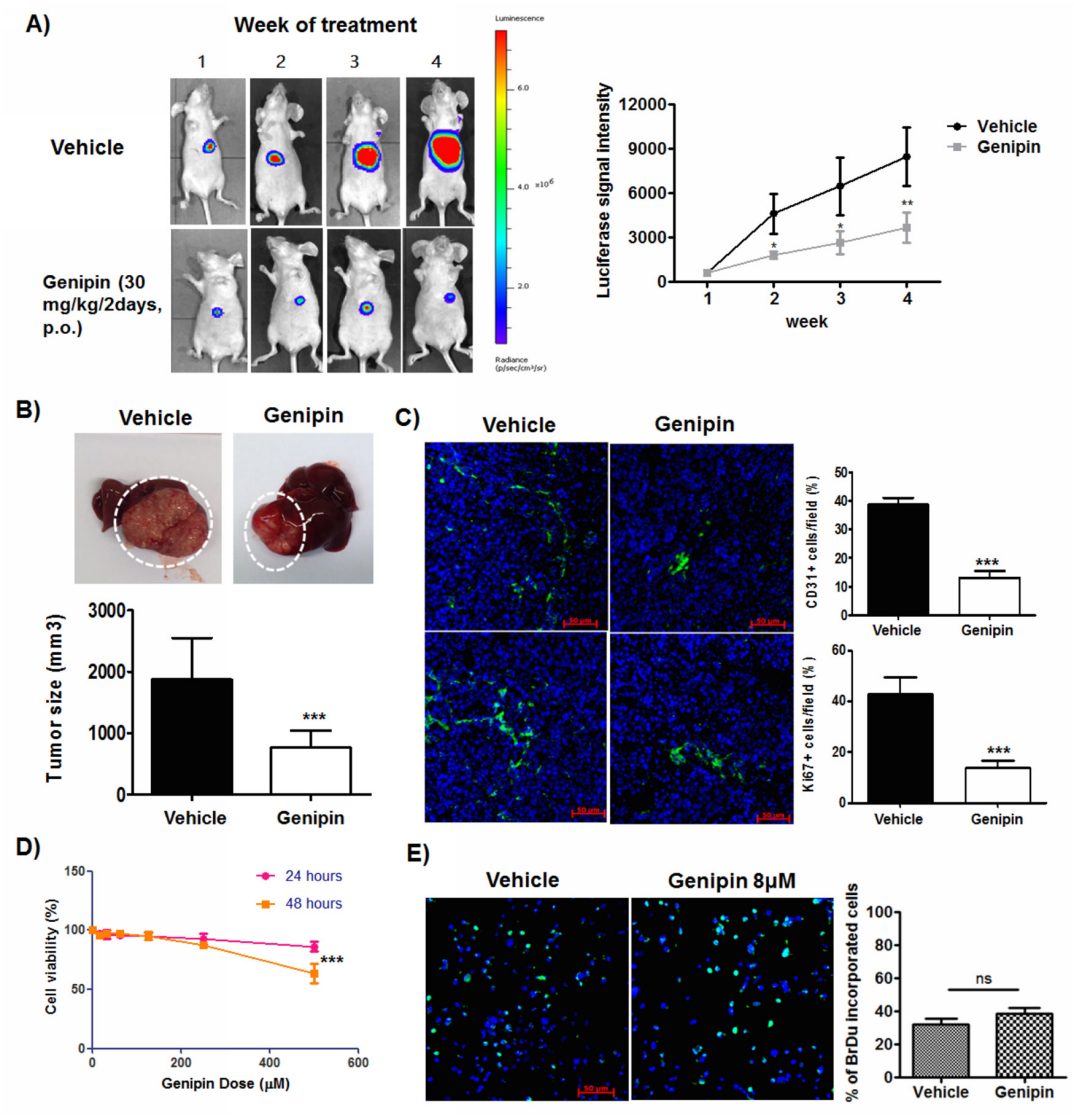
Genipin suppresses orthotopic growth of HCC without induces toxicity to cancer cells **A.** Representative images of orthotopic HCC implanted mice. The tumour growth was monitored every week using luciferase live animal imaging. **B.** Orthotopic HCC tumor growth in mice treated with genipin (n=4) was suppressed. The hepatic tumour size was compared between vehicle and treatment groups. **C.** Immunohistochemistry staining of CD31 and Ki67 of hepatic tumour sections between control and genipin group. The quantitative analysis of the CD31 and Ki67 expressions was performed by calculating the positive stained cells per 40x field of view (Scale bar, 50μm). **D.** Genipin did not cause cell death to Hepa 1-6 up to 250μM after 48 hours of incubation. The percentage of cell viability of Hepa 1-6 cell was determined after treatment with genipin ranging from 0 to 500μM at 24 and 48 hours with MTT assay. **E.** Genipin at 40μM had no direct effect on Hepa 1-6 cell proliferation as evidenced from BrdU assay. Details of the experimental protocols are attached in Materials & Methods. *p<0.05, **p<0.01, ***p<0.001, compared with untreated group.

To systematically examine the inhibitory effect of genipin on hepatocellular carcinoma, we established the orthotopic HCC-implantation mouse model by seeding the subcutaneous-grown HCC tumour cubes onto right lobe of mice liver, as described in our previous studies [18]. It was observed that implanted HCC in mice fed with vehicle (saline buffer) continuously grew during the study, while mice with oral treatment of genipin (30 mg/kg/2days) exhibited slower growth of orthotopic tumour (Figure 1A). The tumour size at the end of 4-week treatment was significantly reduced compared with control group (Figure 1B) while the body weight of mice was not changed (Supplementary Figure S1). There are also no observed pathological changes in gastrointestinal, kidney and lung sections of genipin-intervened mice (Supplementary Figure S2), which indicates that genipin exhibited minimal toxicity to the animals. Genipin reduced CD31- and Ki67-positive cells within HCC, which postulates the suppression of genipin on blood vessel formation and proliferative activity of cancer cells (Figure 1C). Interestingly, we did not observe any direct cytotoxicity of genipin to HCC cells up to 200μM in *in vitro* culture (Figure 1D), nor deceleration of *in vitro* proliferation of HCC cells by genipin treatment (Figure 1E). Our findings may indicate an indirect mechanism underlying the inhibitory effect of genipin on the *in vivo* growth of HCC.

### Genipin suppresses macrophage infiltration in tumour microenvironment of HCC

**Figure 2:**
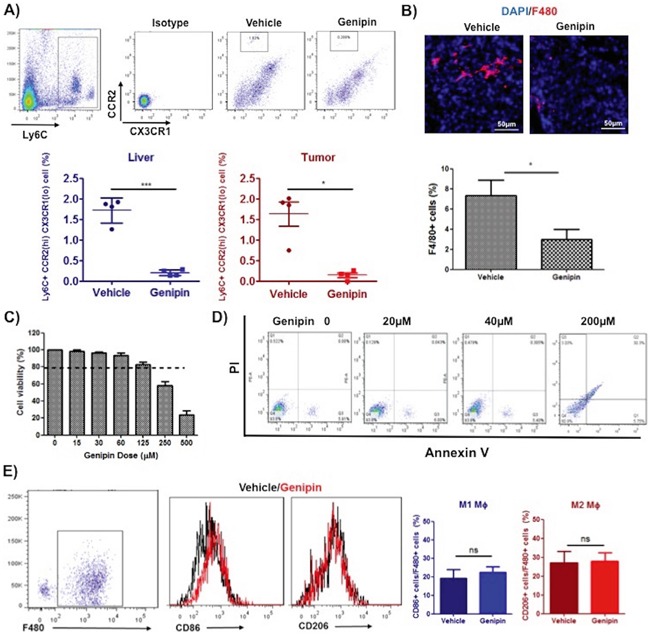
Genipin suppresses TAMs infiltration in tumor microenvironment of HCC **A.** The representative FSC-A/SSC-A plot diagrams of isolated myeloid populations from mice tumour and liver tissues. Antibodies against CCR2 and CX_3_CR_1_ were used to stain cell surface marker of migratory monocytic populations. Ly6C was co-stained to identify monocyte population. Percentage of CCR2 (hi) CX_3_CR_1_ (lo) positive cells infiltrating the liver and tumour are shown (n=4 per group). **B.** Immunohistochemistry staining for F4/80 of frozen tumor tissues after genipin treatment. DAPI was used as nuclear counterstain. *p<0.05, compared with vehicle group (n=4) (Scale bar, 50μm). Genipin did not incur cell death to TAMs up to 200μM as evidenced from **C.** MTT assay and **D.** flow cytometry using Annexin V/7-AAD staining. The detailed protocols for differentiation and culture of TAMs were described in Materials & Methods. **E.** Genipin-treated TAMs showed no difference in CD86/CD206 expression. Antibodies against F4/80 was co-stained for macrophages population and CD86 and CD206 represent the cell surface markers of M1 and M2 phenotype. *p<0.05, **p<0.01, ***p<0.001, compared with untreated group.

Many studies also reported the anti-inflammatory activities of genipin [16, 19]; but whether genipin drives the cross talk between inflammatory cells and cancer cells activities remains unidentified. To assess the effect of genipin on inflammatory cell infiltration into tumour microenvironment, we analysed the expressions of myeloid cellular markers on circulating system and liver tissues of untreated and genipin-treated mice groups. As shown in Figure 2A, the percentage of Ly6C gated CCR2^hi^ CX_3_CR_1_^lo^ migratory monocytic populations was significantly lowered in liver and tumour tissues of mice treated with genipin, whereas there is no changes observed in circulating system (result not shown). Because Ly6C populations enriched on monocytic myeloid lineages, we further detected the expressions of F4/80 macrophage marker in tumour tissues of control and genipin-intervened groups. Fluorescence immunostaining demonstrated that F4/80 stained populations was significantly lowered by genipin treatment (Figure 2B). Considering that deletion of TAMs may be due to the toxic effect of genipin, we first measured if genipin induce macrophage cell death as *in vivo* macrophage population in liver and tumour tissues was lowered in mice treated with genipin. As observed, up to 200μM, genipin exerted no specific cytotoxicity to TAMs (Figure 2C), nor caused apoptosis of TAMs (Figure 2D). Furthermore, differentiating F4/80^+^ macrophages using M1 and M2-like representing cell surface markers showed no differences between CD86^+^ and CD206^+^ populations between control and genipin-treated TAMs (Figure 2E), indicating that genipin-induced anti-inflammatory function is independent to TAMs polarization. Taken together, our observations reveal suppression of TAMs infiltration by genipin without significant alteration in cell viability and phenotype.

### TAMs as a key mediator in inhibition of *in vivo* HCC growth by genipin

**Figure 3:**
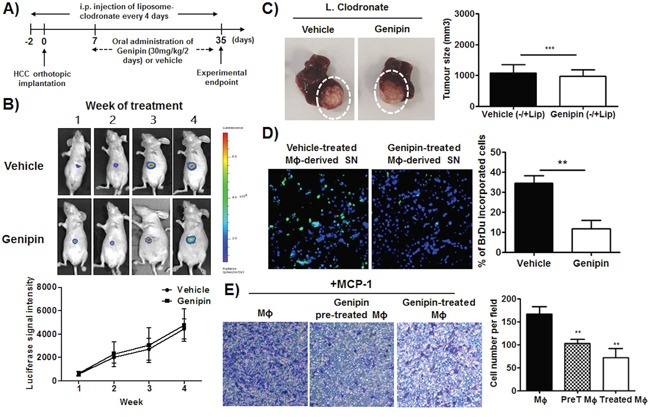
Inhibition of HCC growth by genipin is TAMs-dependent **A.** Schematic diagram of experimental design. **B.** Representative images of orthotopic HCC implanted mice. The tumour growth was monitored every week using luciferase live animal imaging. The luciferase signal intensity was plotted by calculating the mean ± SD (n=4). The details of experimental protocols are attached in Materials & Methods. **C.** Representative image of tumour growth of control and genipin-treated mice after macrophage depletion. The relative tumour volume was calculated after normalization to the tumour size of control and genipin-treated group in the presence of macrophages. **D.** Genipin reduced HCC proliferation as observed from the 5-Bromo-2′-deoxyuridine incorporation assay. TAMs were treated with 40μM genipin for 48 hours and the conditioned media was harvested. Hepa 1-6 cells was cultured in media containing of mΦSN (concentration of 1:5) for 24 hours. BrdU assay was performed using BrdU labelling and detection kit I, and the percentage of BrdU positive labelled cells were counted randomly in 8 microscopic fields per 40 x field of view. (Scale bar, 50μm). **E.** Prior and direct treatment of genipin on TAMs reduced macrophage migration ability. TAMs were pre-treated with genipin 40μm for 48 hours. Chemotaxis assay was performed by seeding the TAMs on culture chamber (8μm) and allow for migration for 16 hours. Soluble MCP-1 (10ng/ml) with or without genipin was added into lower insert. The numbers of crystal violet-stained migratory cells were counted randomly in 5 microscopic fields. *p<0. 05, **p<0. 01, ***p<0.001, compared with untreated group.

To elaborate the relevance of TAMs in genipin-mediated anti-tumour activity, we depleted macrophages using liposome clodronate during the course of genipin administration (Figure 3A). Depletion of TAMs from HCC-bearing mice neutralised the growth inhibition of genipin on orthotopic tumour (Figure 3B). There was no significant difference between the sizes of tumour with or without genipin treatment when TAMs was deleted (Figure 3C), indicating that the inhibitory effect of genipin on HCC growth involves TAMs. This prompted us to further investigate the role of macrophages on HCC behaviour upon genipin treatment. We differentiated the bone marrow-derived monocytic cells using Hepa 1-6 tumour supernatant (TSN), as described in our previous study [18]. The TSN-derived macrophages (TAMs) were cultured with increasing doses of genipin for 48 hours before macrophage supernatant (mΦSN) was harvested. Co-cultured of Hepa 1-6 cells with genipin pre-treated mΦSN reduced Hepa 1-6 proliferation activity as observed from the reduced percentage of BrdU positive Hepa 1-6 cells (Figure 3D). Chemotaxis assay with chemoattractant MCP-1 were carried out to measure the migration of TAMs. TAMs pre-treated with genipin as well as addition of genipin along with MCP-1 demonstrated declined migratory response compared to non-treated group (Figure 3E). These findings suggest that the inhibitory effect of genipin on *in vivo* growth of HCC may be correlated with TAMs activity in tumour microenvironment.

### Genipin reduces TAMs priming in tumour microenvironment

**Figure 4:**
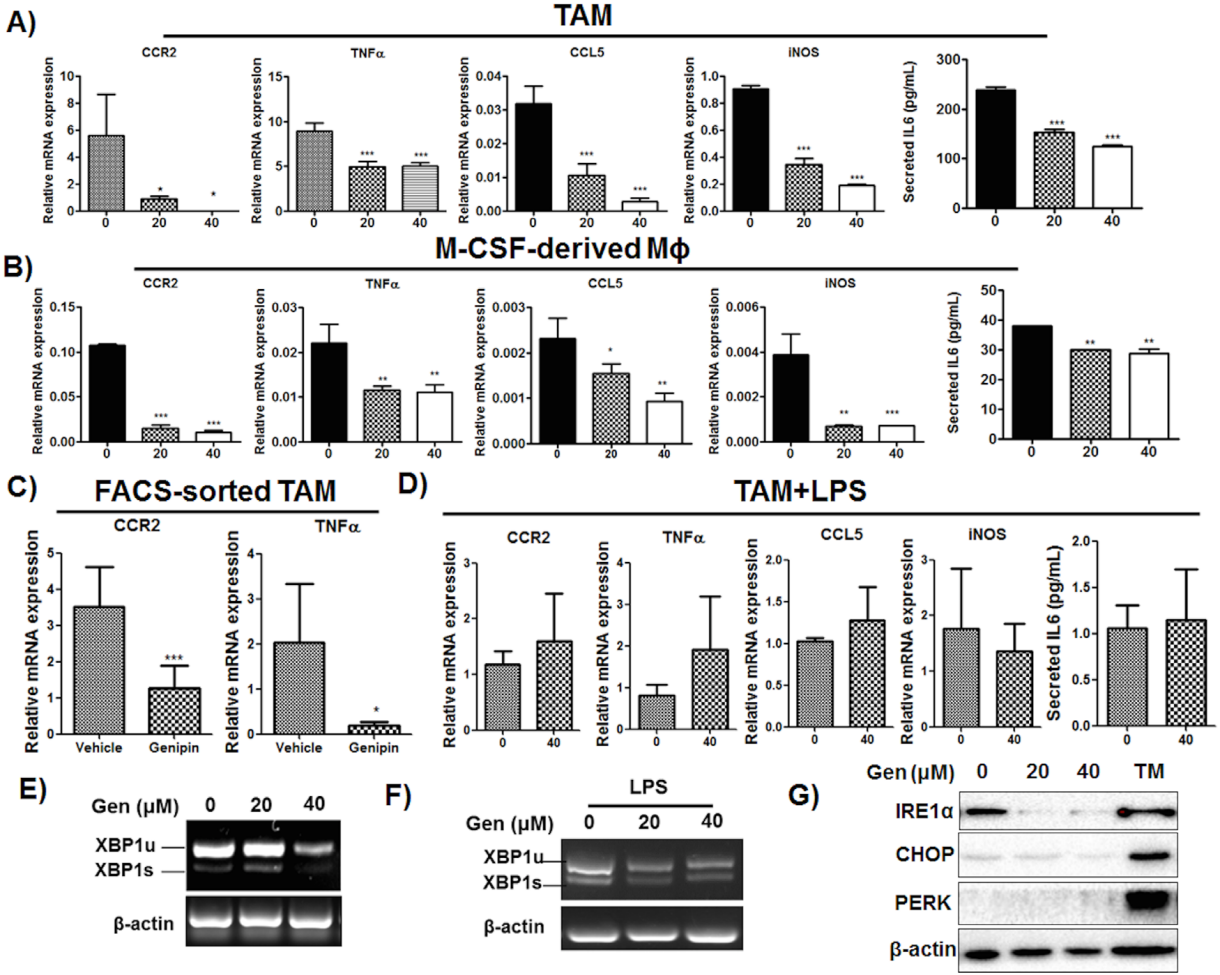
Genipin inhibits activation of TAMs in HCC **A.** The mRNAs profile of TAMs and **B.** M-CSF cultured macrophages after treatment with genipin. Total RNA from cells was harvested and expressions of CCR2, TNFα, CCL5 and iNOS were determined by RT-qPCR. IL6 cytokine secretion from TAMs cultured supernatant and from M-CSF induced macrophages conditioned media. IL6 content was determined with BD CBA kit. The cytokine level was expressed as pg/ml ± SD. **C.** The mRNA expressions of CCR2 and TNFα on FACS-sorted hepatic tumor associated macrophages from control and genipin group. F4/80^+^ macrophages were sorted from hepatic tumors of mice. The mRNA expression was determined by RT-qPCR. Relative fold changes of mRNA levels were calculated after normalization to β-actin. **D.** LPS restored the expressions of CCR2, TNFα, CCL5, iNOS and IL6 in genipin-treated TAMs. The relative fold changes of mRNA levels were calculated after normalization to β-actin. **E.** Reduced XBP1 mRNA splicing was observed in genipin-treated TAMs. Total RNA was extracted from TAMs and the levels of XBP1 mRNA (XBP1u: un-spliced, XBP1s: spliced) was determined by semi-quantitative PCR. β-actin served as loading control. **F.** LPS replenished the genipin-reduced XBP1 mRNA splicing. The XBP1 mRNA expressions of TAMs stimulated with LPS (100ng/ml) after genipin treatment was analysed by semi-qPCR. **G.** The expressions of IRE1α, CHOP and PERK on TAMs after genipin treatment were analysed by immunoblotting. TAMs treated with tunicamycin (10μg/ml) served as positive control. *p<0.05, **p<0.01, ***p<0.001, compared with untreated group.

CCR2 is primarily expressed on macrophages/monocytes and the assembly of CCR2 ligands on mesenchymal stem cells or tumour cells facilitates macrophages recruitment to tumour sites [20]. Blockade of CCL2/CCR2 is associated with reduced influx of inflammatory monocytes and tumour associated macrophages into HCC microenvironment [21]. Initial assembly of TAMs induces expressions of a series of cytokines, which include but not limited to IL6, TNFα and CCL5, further primes the infiltration of monocytes and migration of leukocytes [22]. To examine if genipin reduce the priming process of TAMs within tumour microenvironment, we tested expression of related cytokines in TAMs as well as M-CSF-primed BMDMs. As the mRNA expression of IL6 is non-detectable, we measured its secretion with CBA assay. Genipin significantly decreased expressions of inflammatory mediators CCR2, TNFα, CCL5, iNOS and IL6 on TAMs as well as M-CSF-primed BMDMs (Figure 4A & 4B). On the other hand, genipin did not induce marked changes in expressions of anti-inflammatory factors arginase 1, TGFβ and IL10 (Supplementary Figure S3). Noting IL6 is the primary activator of pro-tumorigenic STAT3 signalling, we also measured STAT3 activity in Hepa 1-6 cells co-cultured with genipin pre-treated mΦSN. We observed decreased phosphorylation of STAT3 while addition of IL6 replenished the effect of genipin on STAT3 inactivation (Supplementary Figure S4). This proposed that IL6 may be one of the responsible cytokines in inhibitory effect of genipin on Hepa 1-6 proliferation. We further isolated F4/80+ TAMs from livers of HCC implanted mice by FACS sorting. Expressions of CCR2 and TNFα were substantially decreased in genipin-intervened group (Figure 4C). Consistently, we detected the expressions of CCR2 and TNFα on hepatic F4/80^+^ macrophages were markedly lower than that from tumour. These results supported the involvement of macrophages in regulating the genipin-mediated tumour suppression. The CCR2 expressions on liver isolated macrophages showed no difference in control and genipin-treated mice; while TNFα was reduced by genipin treatment (Supplementary Figure S5). One of the explanations for this phenomenon is the density of isolated liver macrophages may be highly consisted of Kupffer cells of liver origin. Besides, the reduced expression of related cytokines by genipin was diminished by addition of LPS, a robust activator of TLR4 cascade (Figure 4D). On the other hand, we observed genipin decreased XBP-1 mRNA splicing on TAMs in dose-dependent manner (Figure 4E), while addition of LPS reconstituted spliced XBP-1 (Figure 4F). To determine if reduced XBP-1 splicing led to ameliorated endoplasmic reticulum (ER) stress, we determine expression of ER stress markers CHOP and PERK. As expected, only by addition of tunicamycin, the UPR activator induced activation of ER stress response. Genipin treatment has minimal effect on CHOP and PERK expressions, while their upstream regulator, IRE1α was significantly down-regulated in dose-dependent manner (Figure 4G). Together, these findings suggest that genipin could decrease priming of TAMs in an ER stress-independent manner.

### Inactivation of IRE1α suppresses TAMs priming by genipin

**Figure 5:**
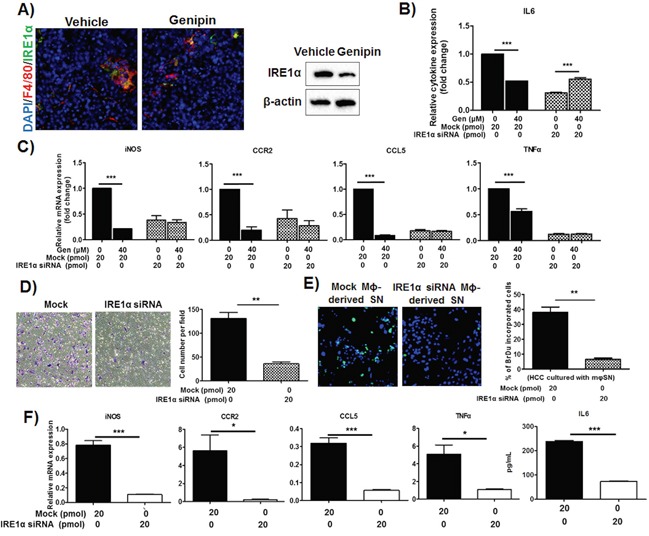
IRE1α mediates inhibition of TAMs activation by genipin in HCC **A.** Treatment of genipin reduced IRE1α expression in hepatic tumour. Antibodies against F4/80 (macrophage marker) and IRE1α were used to co-stain the frozen-sectioned hepatic tumours. DAPI was used as nuclear counterstain (Scale bar, 50μm). Right panel shows the immunoblotting against IRE1α of FACS-sorted TAMs within hepatic tumour of mice with or without genipin treatment. **B.** The reduced IL6 cytokine levels by genipin was normalized by RNA interference against IRE1α. RNA interference was performed as described in Materials & Methods. The relative cytokine content was calculated after normalization to the cytokine expression of untreated group. The same also observed in **C.** mRNAs expressions of genipin-treated TAMs with IRE1α silencing. The relative mRNAs expression was calculated after normalization to the expression of untreated group. **D.** TAMs with IRE1α silencing had diminished effect on migration property. **E.** Reduced Hepa 1-6 cell proliferation was observed on cells cultured in media containing supernatant of macrophages with IRE1α silencing. **F.** The inflammatory genes mRNAs expressions and IL6 secretion were reduced in TAMs with IRE1α silencing. *p<0. 05, **p<0. 01, ***p<0.001, compared with untreated or mock group.

Recent study reported that activation IRE1α, a conserved sensor of unfolded protein responses is responsible on the induction of inflammatory cytokines by macrophages [23]. Consistently, we observed the genipin treatment reduced IRE1α protein expression on F4/80+ macrophages in liver tissue of mice (Figure 5A), which suggested IRE1α may be the mediator of genipin-induced inhibition of inflammatory response. To verify the hypothesis, we used RNA interference against IRE1α to silent the gene expression on TAMs before intervention of genipin. It was accompanied with increased IL6 production and normalization of relative inflammatory gene expressions between the groups with or without genipin (Figure 5B and 5C). As shown in Figure 5D, silencing of IRE1α reduced the migratory property of macrophage. Culturing of Hepa 1-6 cells with IRE1α-silencing TAMs-containing medium also reduced the Hepa 1-6 proliferation (Figure 5E). This notion is further supported by our observations that the responsible inflammatory genes was blocked by IRE1α silencing (Figure 5F). Furthermore, we observed the IRE1α highly expressed in 7-days differentiated BMDMs with TSN, while immature BMDMs at earlier phase expressed much lower IRE1α (Supplementary Figure S6). This further reveals IRE1α as a crucial factor for the macrophage priming process. Taken together, these results confirmed that IRE1α is involved in genipin-correlated inhibitory effects on macrophage migration and HCC proliferation.

### IRE1α is involved in genipin-correlated inactivation of NF-κB via dissociation of IKK complex

**Figure 6:**
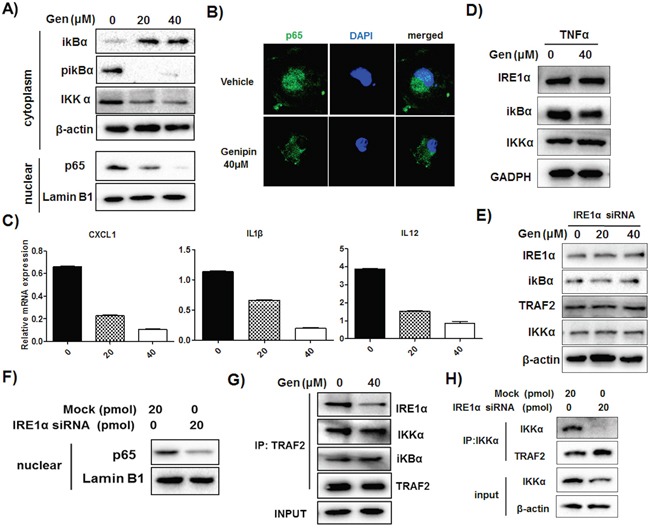
Reduced association of IRE1α-TRAF2-IKK may be responsible for genipin-regulated inactivation of NF-κB **A.** TAMs were treated with 20μM and 40μM genipin for 48 hours. Expressions of IκBα, p-IκBα and IKKα of total extract and p65 in nuclear extract were determined by immunoblotting. Lamin B1 served as nuclear loading control. **B.** Genipin reduced p65 nuclear translocation on TAMs. Cells were stained with p65 antibody and counterstained with DAPI, followed with visualization under confocal microscope (magnification: 63x). **C.** Expressions of p65/p50 specific target genes on TAMs after genipin treatment were analyzed by RT-qPCR. **D.** TAMs were treated with genipin in the presence of soluble TNFα (10ng/ml). Expressions of IREα, IκBα, and IKKα were determined. **E.** TAMs with RNA interference against IRE1α were treated with genipin. Expressions of IREα, IκBα, TRAF2 and IKKα were determined. **F.** Silencing of IREα reduced p65 nuclear translocation in TAMs. Expression of p65 in nuclear extract of TAMs was determined. **G.** The interactions of IREα with IκBα, TRAF2 and IKKα were determined by co-immunoprecipitation assay and immunoblotting. TRAF2 was immune-precipitated and expressions of interacted proteins were normalized by β-actin as input. **H.** IKKα protein on wild type and IRE1α silencing TAMs was immune-precipitated; the expressions of TRAF2, IKKα and IRE1α (not detected) were determined. *p<0. 05, **p<0. 01, ***p<0.001, compared with untreated group.

We observed that genipin induced IκBα accumulation and reduced its phosphorylation, down-regulated IKKα and blocked the translocation of p65 into nuclear regions of TAMs (Figure 6A & 6B). This was accompanied with reduced activation of p65/p50-specific target genes CXCL1, IL1β and IL12 (Figure 6C). Addition of TNFα, the NF-κB inducer, replenished genipin-induced IκBα accumulation (Figure 6D). These results, together with abolishment of decreased inflammatory gene expressions induced by genipin after intervention of TNFα (Supplementary Figure S7), indicate that genipin is a potent NF-κB inhibitor. Further investigation of the correlation between IRE1α and NF-κB signaling showed that RNA interference against IRE1α abolished the inhibition of NF-κB by restoring the NF-κB related protein expressions to basal levels (Figure 6E). IRE1α depletion also blocked the p65 nuclear translocation (Figure 6F) and replenished the reduced expression of p65/p50-transactivated genes in the presence of genipin (Supplementary Figure S8). Noting that TRAF2, the adaptor molecule of NF-κB, may bridge between IRE1α and NF-κB signaling, we performed co-immunoprecipitation assay with TRAF2 antibody on TAMs. It was observed that there is association between TRAF2 and IKKα, IκBα as well as IRE1α (Figure 6G). While TRAF2 expression had minimal change, the reduced IRE1α and IKKα may further reduce the association of IRE1α-TRAF2-IKKα by genipin, which would lead to inhibition of p65/p50. Surprisingly, we couldn't detect IRE1α during co-immunoprecipitation of IKKα, suggested that IKKα has no direct interaction with IRE1α. Inactivation of IRE1α dissociated the interaction of TRAF2 and IKKα as observed from diminished IKKα when IRE1α is deleted, further evidenced that IRE1α as an essential mediator in association of IKKα and TRAF2 (Figure 6H). Taken together, our results postulated that inactivation of IRE1α reduced the association of IKKα and TRAF2. The activity further led to stabilization of IκBα and inhibition of p65 nuclear translocation.

## DISCUSSION

Chronic inflammation is one of the important factors of carcinogenesis, in which the inflammatory mediators such as TNFα, IL-1β and IL-6 secreted by macrophages promote tumour growth by deregulating the cell cycle checkpoints and cell repair machinery [5]. Although recent study classify TAMs into M1 and M2 phenotypes and high proportions of pro-inflammatory M1-like macrophages is associated with tumour growth regression [24], it is still controversial whether inflammation-favourable environment is promising therapeutic strategy for cancer therapy. Indeed, some studies have reported that the reduced inflammation-associated macrophages inhibited HCC development, which is independent to M1/M2 programming [25]. Moreover, IL6 produced by TAMs promoted cancer stem cell expansion and the expression level is proportional to the HCC clinical stages [26]. This further indicates that attenuation of inflammation independent to macrophage phenotype skewing may also be beneficial for HCC treatment. Indeed, our study observed reduction of macrophage priming by genipin with reduced secretion of pro-inflammatory cytokines. This is associated with reduced secondary infiltration of inflammatory monocytes and TAMs into tumour microenvironment. Besides, we also found that treatment of macrophage scavenger, liposome clodronate unexpectedly led to reduced growth of HCC, which was consistent with the observations in other previous studies [27, 28]. This indicates that removal of TAMs from tumour microenvironment regardless its phenotype may render tumour inhibition. Some “anti-macrophage” treatments are currently under investigation. Investigational or approved antagonists of TAM-favouring factors, such as CCL2 and CCL5, have been proved to be associated with therapeutic potential [29]. Our findings on the inhibitory effect of genipin on TAMs priming and infiltration, postulated the evidence of inflammation suppressor in anti-cancer treatment.

Accumulating studies have demonstrated the role of IRE1α in modulating the pathogenesis of inflammation associated diseases such as inflammatory arthritis, diabetes and dyslipidaemia [23, 30, 31]. Most of the time, activated IRE1α induces unconventional splicing of 26-nucleotide stretch from the mRNA encoding X-box-binding protein 1 (XBP-1), resulted in encoding of spliced XBP-1, that translocate to nucleus for initiation of unfolded protein response (UPR) related genes transcription [32]. In addition to the conventional ER signal sensing, recent studies have extended the role of IRE1α-XBP-1 axis to modulation of metabolic homeostasis and immune response [33, 34]. Previous study showed that the engagement of toll like receptors to IRE1α induced XBP-1 splicing on macrophages leading to enhanced production of IL6 and TNFα independent to ER stress [33]. In consistent with our study, we also observed increased XBP-1 processing on TSN induced macrophages and addition of genipin blocked XBP-1 cleavage without incurring any stress response on macrophages. Although we did not perform XBP1 deletion, our findings evidenced that IRE1α-XBP1 axis is likely involved in genipin-repressed inflammatory response. First, genipin down-regulated the expressions of IL6 and TNFα, the mature XBP1 trans-activated genes. Second, intervention of LPS, the TLR4 agonist reconstituted the spliced XBP1 and increased the levels of IL6 and TNFα, rather than normalize them.

**Figure 7:**
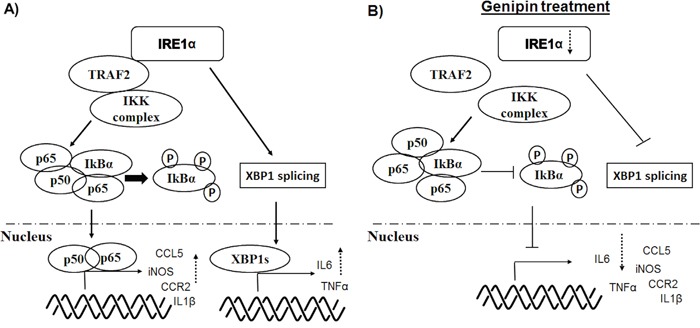
Schematic diagram of the mechanisms underlying inhibition of TAMs by genipin

IRE1α also mediates inflammation through engagement with several downstream effectors [31, 35]. Previous study suggested that XBP1 deletion may not sufficient in blocking the broad spectrum of inflammatory genes expression [23]; and consistently, our study demonstrated that IRE1α deletion normalized a group of inflammatory genes apart from XBP-1 regulated IL6 and TNFα. The cytoplasmic kinase domain of activated IRE1α interacts with JNK and IκB kinase (IKK) via adaptor protein TRAF2, and the binding is likely determined by the degree of IRE1α oligomerization [36]. In our study, we did not observed reduction of TRAF2, the adaptor protein of IRE1α. The inhibitory effect of IRE1α suppression by genipin on downstream NF-κB machinery may be therefore due to dissociation of IRE1α from TRAF2 binding. Indeed, previous study demonstrated that IRE1α forms complex with IKK through TRAF2 and the activity further leads to phosphorylation of IκBα and NF-κB activation [37], indicated that IRE1α is the essential mediator for interaction between IKK and TRAF2. Reduction of IRE1α by genipin therefore abrogated the association of IKK and TRAF2, which as a result stabilized IκBα and blocked the nuclear translocation of p65/p50 (Figure 7).

In conclusion, our results demonstrated that the tumour suppressive effect of genipin was mediated by TAMs. Genipin potently inhibited *in vivo* orthotopic growth of HCC and significantly reduced infiltration of inflammatory monocytes and TAMs in HCC microenvironment. Genipin suppressed migration of TAMs and expression of macrophage priming-related cytokines. Inhibition of TAMs priming by genipin was correlated with its effect in specifically deletion of IRE1α in TAMs. Regulation of genipin on TAMs involved inactivation of XBP-1 splicing and NF-κB machinery. The study suggests genipin, aiming at IRE1α inactivation may represent a promising immunomodulatory therapeutic candidate for hepatocellular carcinoma.

## MATERIALS AND METHODS

### Animal studies

The detailed animal model establishment has been reported in our previous study [18]. Luciferase-tagged HCC cells-constituting tumor were cut into 1mm^3^ before implanted into the left lobe of mice liver. After one week of laparotomy, the growth of liver tumor was examined via luciferase imaging analysis. All tumor-presenting mice were then orally given genipin (30 mg/kg/2days) or equal volume of PBS (n=4) for 5 weeks. Liver tumor growth was monitored weekly. Macrophage depletion was performed as indicated in Figure 3A. Briefly, mice in macrophage removal groups were intraperitoneally administrated with clodronate liposome (0.1ml/10g, ClodronateLiposomes.com, Netherland) every 4 days along with genipin intervention. Animal experimental protocols were approved by the Committee on the Use of Live Animals in Teaching and Research (CULATR) of The University of Hong Kong, Hong Kong.

### Fluorescence activated cell sorting (FACS)

Liver and tumor tissues were minced and harvested with collagenase solution at 37°C for 30 minutes. The pellets were re-suspended in RPMI 1640 and mononuclear cells were separated according to Percoll density gradient centrifugation procedure. Cells were washed with PBS and stained with anti-mouse CCR2, CX3CR1, Ly6C, F4/80, CD86 and CD206 antibody for 15 minutes. Corresponding isotypes antibodies were used as controls. The FACS analysis was performed on FACS Canto II cytometer (BD) and cell sorting on FACS Aria I cytometer (BD). Data were analyzed with FlowJo software.

### Immunohistochemistry staining

Tumor tissues were fixed with 4% paraformaldehyde and then 30% sucrose solution. Frozen sections were cut and incubated with CD31, Ki67 and F4/80 antibodies. The stained slides were visualized under fluorescent microscope.

### Isolation of bone marrow derived mononuclear cells and macrophage migration

Bone marrow derived mononuclear cells (BMDMs) were harvested from femurs and tibias of 6- to 8-week-old C57BL/6 mice and cultured with complete RPMI-1640 (supplemented with 10% Fetal Bovine Serum and 1% Penn/Strep). The differentiation of mononuclear cells to macrophages was performed either by culturing the isolated BMDMs with murine M-CSF (10ng/mL) or 30% tumor cell culture supernatant for 7 days. The detailed procedures are described in our previous study [18]. Migration assay was performed through seeding the macrophages on receiving insert (8μm, Corning, USA) and allowed to migrate for 16 hours. The chambers were then removed and the cells under chamber were fixed in 4% paraformaldehyde. The fixed cells stained with crystal violet for 2 hours before visualized by inverted microscopy.

### Co-culture of HCC cells with macrophage supernatant

The macrophage-conditioned medium was harvested after the macrophages incubated with genipin for 48 hours. The Hepa 1-6 cells were cultured in 20% macrophage supernatant (1:5 final dilution) and incubated for 24 hours. Cell proliferation activity was measured by 5-Bromo-2′-deoxyuridine labelling and detection kit I (Roche), according to manufacturer's instructions. The image was captured using fluorescent microscopy.

### Western blotting

Total protein was extracted from HCC and macrophage cells via RIPA lysis buffer supplemented with cocktail proteinase inhibitor and phosphatase inhibitor (1mM Na_3_VO_4_ and 1mM NaF). Nuclear extraction was performed using nuclear extraction buffer (ThermoFisher). The protein lysates were separated on SDS-PAGE gel and blotted onto polyvinylidene difluoride membranes. The membrane was incubated with IRE1α, CHOP, PERK, TRAF2, IKKα, IκBα, and β-actin. The blots were labeled with HRP–labelled secondary antibody before subjected to chemiluminescence analysis.

### Quantitative real time PCR

Total RNA was isolated with TRIzol reagent (Invitrogen) and cDNA was synthesized with PrimeScript RT Reagent kit (TaKaRa), according to manufacturer's instructions. Real time PCR was performed using SYBR Green Master mix (TaKaRa) and 1μM primers using Light Cycler 480 PCR system (Roche, USA). The murine β-actin serves as endogenous control and primers sequences are listed in Supplementary Table S1.

### RNA interference

The macrophage cells were incubated with RPMI 1640 without antibiotics that pre-mixed with 10μM of Lipofectamine RNAiMAX (Invitrogen) and 1nmol of siRNA (FlexiTube, Qiagen), according to manufacturer instructions. The transfection efficiency was assessed by immunoblotting assay.

### Immunofluorescence staining

Prior to immunofluorescence staining, macrophages cells were grown on glass coverslip and cultured with or without genipin. After fixing the cells with 4% paraformaldehyde, the cells were stained with p65 antibody and counterstained with DAPI. Specimen was mounted and examined by fluorescence confocal microscopy (Carl Zeiss LSM780).

### Co-immunoprecipitation assay

The assay was performed using protein G magnetic beads (Pureproteome Millipore), based on manufacturer instructions. The target protein was precipitated with antibody that pre-bound with magnetic beads, followed with elution with PBS in 0.1% Tween 20. The protein lysates were separated on SDS-PAGE and visualized using chemiluminescence system.

### Statistical analysis

Data were analyzed as mean value ± SD and Student's T-test or two-way ANOVA was used to assess the result significance. A p value < 0.05 was deemed as statistically significant.

## SUPPLEMENTARY FIGURES AND TABLE


