# Supplementary material for: IRE1α inhibition by natural compound genipin on tumour associated macrophages reduces growth of hepatocellular carcinoma
Source: Oncotarget. 2016 May 30;7(28):43792–804. doi: 10.18632/oncotarget.9696 (PMC5190060; doi:10.18632/oncotarget.9696)
Supplement: Supplementary file 1 [file oncotarget-07-43792-s001.pdf]

IRE1α inhibition by natural compound genipin on tumour associated macrophages reduces growth of hepatocellular carcinoma

SUPPLEMENTARY FIGURES AND TABLE

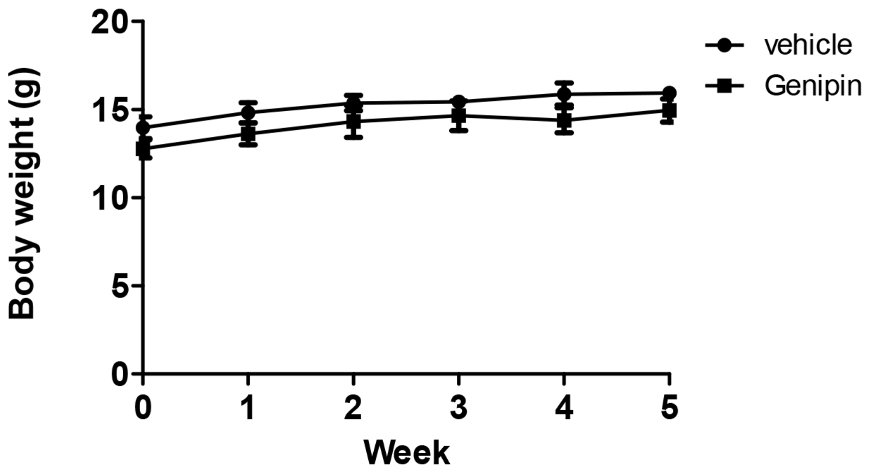

Supplementary Figure S1: Body weight of orthotopic HCC implanted mice. The detailed animal model establishment is described in Materials & Methods. Body weight of mice was measured every week. There are no significant differences in body weight between control and treatment groups.

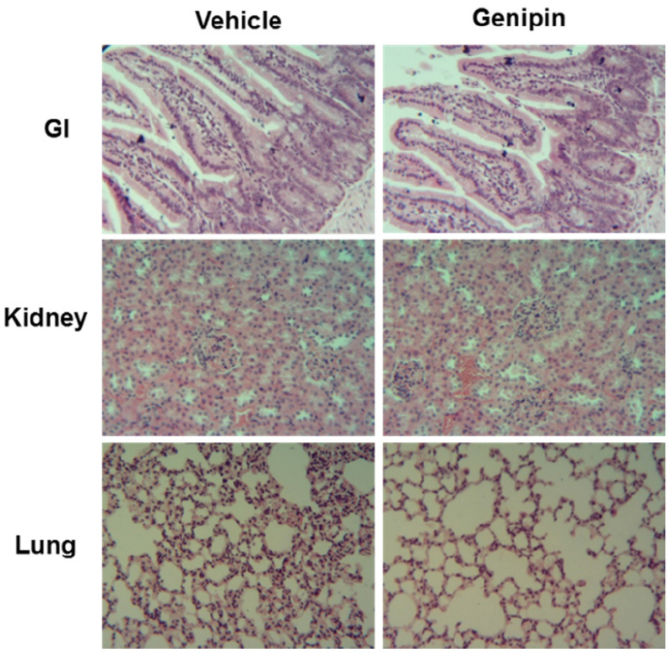

Supplementary Figure S2: Haematoxylin-Eosin staining of different part of tissues. The gastrointestinal, kidney and lung tissues of mice were harvested and fixed in 4% parafolmadehyde. Paraffin sections were cut and stained with H&E. The stained slides were visualized under microscope (magnification: 10x). There are no significant pathological changes in GI, kidney and lung across the groups.

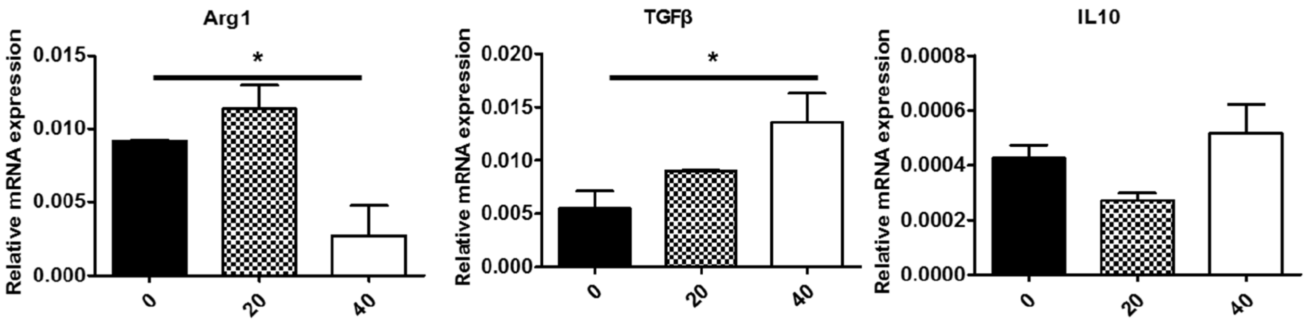

Supplementary Figure S3: The M2 mRNAs expressions of TAMs. TAM was treated with vehicle or 20μM and 40μM genipin for 48 hours and mRNA expressions of Arg1, TGFβ and IL10 were measured by qPCR. There are no obvious expression changes in genipin-treated TAMs was observed.

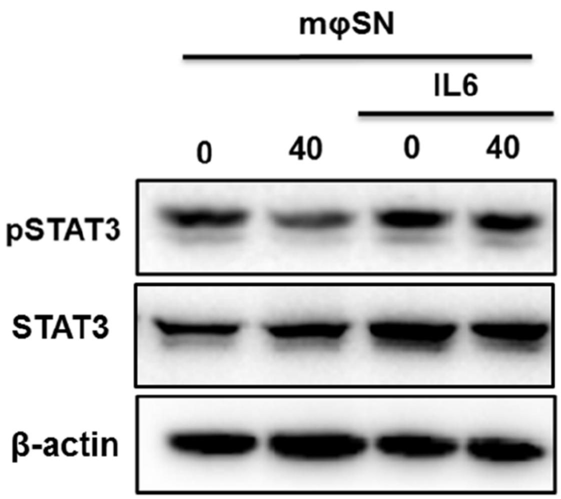

Supplementary Figure S4: Expressions of pSTAT3 and STAT3 of HCC cells. Hepa 1-6 cells co-cultured with vehicle and genipin pre-treated macrophage supernatant (mΦSN). The expressions of phosphorylated STAT3 and STAT3 were detected by immunoblotting. The pSTAT3 expressions of Hepa 1-6 cells reduced upon cultured in genipin-pre-treated macrophage supernatant; while IL6 replenished the effect of genipin on STAT3 dephosphorylation.

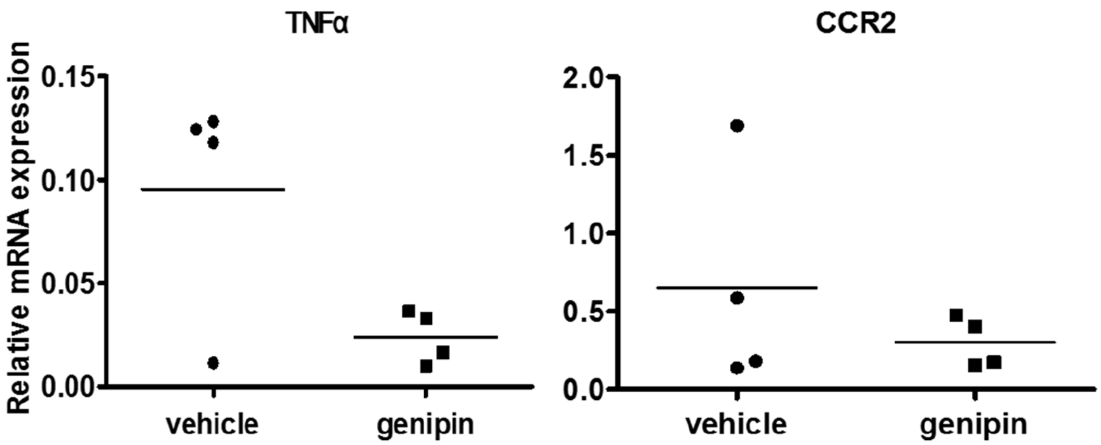

Supplementary Figure S5: mRNAs expressions from liver isolated F4/80+ macrophages. F4/80+ macrophages were sorted from mice livers. The expressions of CCR2 and TNFα were detected by RT-qPCR. The CCR2 expressions on liver isolated macrophages showed no difference in control and genipin-treated mice; while TNFα was reduced by genipin treatment.

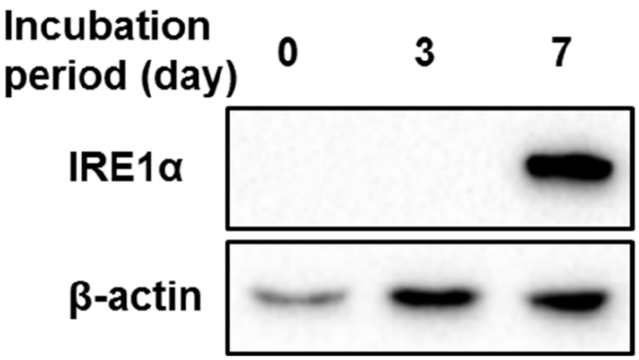

Supplementary Figure S6: Expression of IRE1α of bone marrow derived macrophage. Bone marrow derived monocytic cells were differentiated in Hepa 1-6 derived tumour supernatant (TSN) for 0, 3 and 7 days of incubation. The expressions of IRE1α was detected by immunoblotting. The IRE1α expression of macrophage cells increased after 7 days of culture with TSN, while it is not detected in early maturation state.

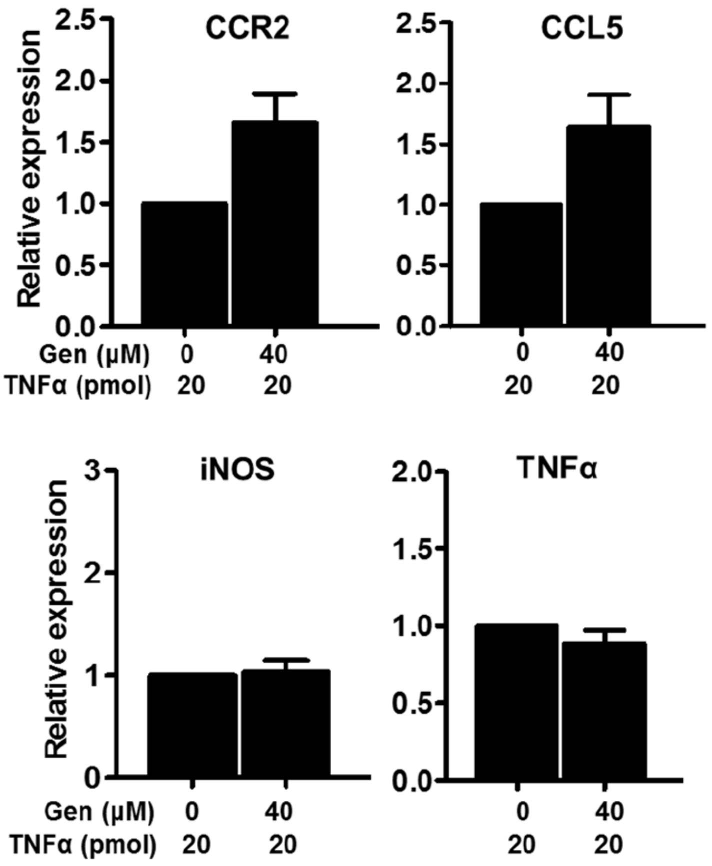

Supplementary Figure S7: The mRNAs expressions of TAMs stimulated by TNFα. TAM was treated with vehicle and 40μM genipin for 48 hours and soluble TNFα was added for 6 hours before cells were harvested. The mRNA expressions of CCR2, CCL5, iNOS and TNFα were measured by qPCR. TNFα normalized the decreased expressions of inflammatory genes induced by genipin.

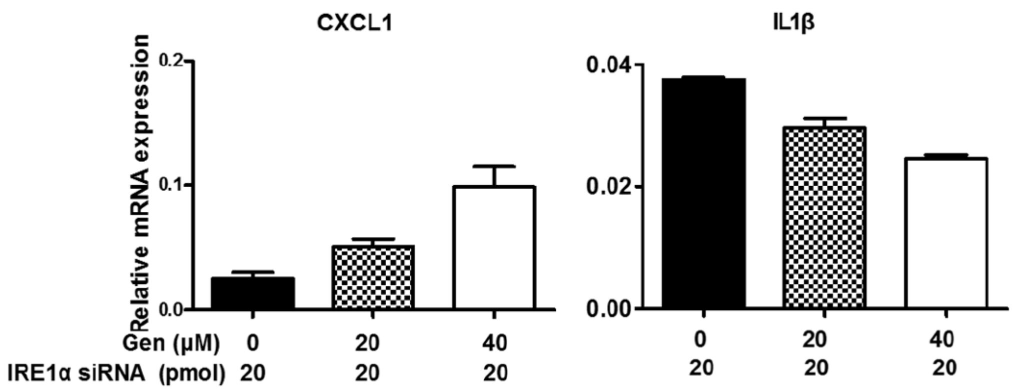

Supplementary Figure S8: The p65/p50 transactivated genes expressions. TAM with RNA interference against IRE1α was treated with vehicle and genipin for 48 hours. The mRNA expressions of p65/p50 target genes, CXCL1 and IL1β were measured by qPCR. IRE1α deletion replenished the reduced expression of p65/p50-transactivated genes by genipin.

Supplementary Table S1: Sequence of mouse primer pairs used

Gene name	Forward	Reverse
CCR2	5’-GTGAGCAGGAAGAGCAGGTC-3’	5’-CCTGCAAAGACCAGAAGAGG-3’
TNFα	5’-CTGTAGCCCACGTCGTAGC-3’	5’-TTGAGATCCATGCCGTTG-3’
CCL5	5’-GTGCCCACGTCAAGGAGTAT-3’	5’-AGCAAGCAATGACAGGGAAG-3’
iNOS	5’-GTTCCTCAGCCCAACAATACAAGA-3’	5’-GTGGACGGGTCGATGTCAC-3’
CXCL1	5’-GCAGACCATGGCTGGGATT-3’	5’-TGTCAGAAGCCAGCGTTCAC-3’
IL1β	5’-GTGTGGATCCAAAGCAATAC-3’	5’GTCTGCTCATTCATGACAAG-3’
IL12	5’-TACTAGAGAGACTTCTTCCACAACAAGAG-3’	5’-TCTGGTACATCTTCAAGTCCTCATAGA-3’
β-actin	5’-CCTGAGGCTCTTTTCCAGCC-3’	5’-TAGAGGTCTTTACGGATGTCAACGT-3’
